# Acceptability and feasibility of a low-cost, theory-based and co-produced intervention to reduce workplace sitting time in desk-based university employees

**DOI:** 10.1186/s12889-015-2635-z

**Published:** 2015-12-24

**Authors:** Kelly Mackenzie, Elizabeth Goyder, Francis Eves

**Affiliations:** School of Health and Related Research (ScHARR), University of Sheffield, Regent Court, 30 Regent Street, Sheffield, S1 4DA UK; The School of Sport, Exercise and Rehabilitation Sciences, College of Life and Environmental Sciences, University of Birmingham, Edgbaston, Birmingham, B15 2TT UK

**Keywords:** Sitting, Sedentary, Workplace, Office, Occupation, Desk-based, Employees, Staff

## Abstract

**Background:**

Prolonged sedentary time is linked with poor health, independent of physical activity levels. Workplace sitting significantly contributes to sedentary time, but there is limited research evaluating low-cost interventions targeting reductions in workplace sitting. Current evidence supports the use of multi-modal interventions developed using participative approaches. This study aimed to explore the acceptability and feasibility of a low-cost, co-produced, multi-modal intervention to reduce workplace sitting.

**Methods:**

The intervention was developed with eleven volunteers from a large university department in the UK using participative approaches and “brainstorming” techniques. Main components of the intervention included: emails suggesting ways to “sit less” e.g. walking and standing meetings; free reminder software to install onto computers; social media to increase awareness; workplace champions; management support; and point-of-decision prompts e.g. by lifts encouraging stair use. All staff (*n* = 317) were invited to take part. Seventeen participated in all aspects of the evaluation, completing pre- and post-intervention sitting logs and questionnaires. The intervention was delivered over four weeks from 7th July to 3rd August 2014.

Pre- and post-intervention difference in daily workplace sitting time was presented as a mean ± standard deviation. Questionnaires were used to establish awareness of the intervention and its various elements, and to collect qualitative data regarding intervention acceptability and feasibility.

**Results:**

Mean baseline sitting time of 440 min/workday was reported with a mean reduction of 26 ± 54 min/workday post-intervention (*n* = 17, 95 % CI = −2 to 53). All participants were aware of the intervention as a whole, although there was a range of awareness for individual elements of the intervention. The intervention was generally felt to be both acceptable and feasible. Management support was perceived to be a strength, whilst specific strategies that were encouraged, including walking and standing meetings, received mixed feedback.

**Conclusions:**

This small-scale pilot provides encouragement for the acceptability and feasibility of low-cost, multi-modal interventions to reduce workplace sitting in UK settings. Evaluation of this intervention provides useful information to support participatory approaches during intervention development and the potential for more sustainable low-cost interventions. Findings may be limited in terms of generalisability as this pilot was carried out within a health-related academic setting.

## Background

In recent years, a new research paradigm has emerged, highlighting the potential deleterious health impacts of sustained sedentary behaviour [1], independent of the amount of physical activity undertaken [2, 3]. Potential health consequences include an increased risk of: cardiovascular disease [4–6]; metabolic syndrome/type 2 diabetes [4, 6–9]; obesity [10]; hypertension [11]; some cancers [12]; depression [13]; and musculoskeletal problems [14]. Furthermore, sustained sedentary behaviour has shown to be independently associated with an increased risk of premature mortality [6, 9, 15]. As a result of these associated health impacts, reducing the amount of time adults spend being sedentary has been identified as an important public health initiative [16].

Sedentary behaviour is defined as any waking behaviour characterised by an energy expenditure ≤1.5 Metabolic Equivalents (METs) while in a sitting or reclining posture [17], and is distinct from light intensity activities such as standing and walking, which have been shown to confer some health benefits [18, 19]. As it is impractical to measure energy expenditure in the majority of studies, sedentary behaviour can be most simply operationalised as sitting time [20].

Prolonged workplace sitting is especially significant when considering interventions to reduce sitting time due to the increase in desk-based jobs over recent decades [21] and the fact adults spend approximately 60 % of their waking hours in the workplace [22, 23]. Furthermore, recent evidence has shown that office workers sit for an average of six hours in an eight-hour working day and sitting is most often accumulated through prolonged, unbroken bouts [24, 25]. Observational studies looking at office work and sitting time have shown a positive association, which significantly contributes to overall daily sitting time [24, 26]. It is this recognition of the importance of sitting in the workplace which has highlighted the need for the development of interventions to reduce workplace sitting [27].

This paper describes the development and mixed methods evaluation of an intervention to reduce workplace sitting time, drawn from a four week uncontrolled study, which is intended to be a precursor to a larger controlled trial. The main aim of this study was to explore intervention acceptability and feasibility, which was addressed by documenting: pre- and post-intervention changes in daily sitting time; participants’ awareness of the various elements of the intervention; and participants’ views of the intervention. It also provides initial evidence on effectiveness of low-cost interventions, which could be used to inform future workplace studies.

## Methods

### The intervention

The development of the intervention was based on three stages as highlighted by Neuhaus et al. [28]: conceptualisation (assessing the evidence-base and providing theoretical grounding); formative research (a participative approach with the target audience to engage and promote “buy-in”); and finally pilot testing (to establish the intervention’s acceptability, feasibility and impact using mixed methods).

#### Conceptualisation

Many of the interventions that have been developed to reduce workplace sitting have focused on a single-level of influence, such as the provision of educational sessions/behaviour change support [29] or environmental/ergonomic modifications to the workplace [30–32]. However, Gilson et al. [33] explored employees' perceptions of sedentary behaviour and strategies to reduce workplace sitting, which suggested that, in order to have the greatest impact on a reduction in workplace sitting time, interventions should address multiple levels of influence, such as individual-, social-, organisational- and environmental-levels.

Theoretical support has been identified as an essential element in the development of complex interventions [34, 35]. A theoretical framework commonly used in physical activity interventions [36], which encompasses these multiple levels of influence, is the socio-ecological model (SEM) [37, 38]. It has therefore been suggested that SEM could be utilised to theoretically underpin interventions targeting workplace sitting [39, 40]. Studies that have developed and evaluated multi-modal interventions, which integrated behaviour change, social influences and environmental modifications are now emerging [28, 41–43].

Many of the studies that aimed to reduce workplace sitting time included the use of ergonomic adaptations such as treadmill workstations [44, 45], cycling workstations [46], portal pedal machines [32, 42], and the use of sit-to-stand adjustable desks [41, 43], which, when part of a multi-modal intervention, consistently contributed to a significant reduction in workplace sitting. Nevertheless, there is a need to develop pragmatic, low-cost interventions to encourage greater uptake amongst employers, for whom the cost of these relatively expensive interventions may be a significant barrier to implementation.

Potentially low-cost elements of an intervention may include the use of reminders, prompts and weekly emails. The installation of free computer software reminding staff to take regular breaks, has been shown to produce a significant reduction in workplace sitting when compared to controls [47]. In addition, a systematic review looking at point-of-decision prompts to encourage stair use suggested there is strong evidence to support this type of intervention [48], which have also been effective in reducing workplace sitting [29, 47, 49, 50]. In a randomised control trial, weekly emails containing motivational messages and suggestions to break-up sitting time, such as lunchtime walks and incidental walking (e.g. reducing the use of email/telephone in favour of face-to-face contact) were shown to have the greatest impact on sitting time [51].

#### Formative research

Eleven staff from the School of Health and Related Research (ScHARR), an academic school focusing on health-related research in the University of Sheffield, UK, volunteered to be part of an intervention development focus group, of which seven were able to attend a one-hour session. The framework for this session was taken from previous work by Dunstan et al. [52] and involved an initial description of the associations between prolonged sitting and health, followed by a "brainstorming" session where strategies were identified by participants on how to reduce workplace sitting time. The four participants who were unable to attend the meeting submitted suggestions via email.

### Study design

An uncontrolled pre-post intervention design was used with a single sample and two data collection time points over the five week study period. All study procedures were undertaken by a student undertaking a Masters in Public Health. Ethical approval was provided by the ScHARR Ethics Committee (reference number 0745/KW 30). Intervention development focus group participants provided informed written consent, for other participants, informed consent was implied by completion and return of questionnaires and sitting logs.

### Procedure

#### Eligibility criteria

All employees (*n* = 317) of ScHARR were eligible to participate in this study. Employees come from four Sections within the school: Public Health (PH), Health Services Research (HSR), Health Economics and Decision Science (HEDS) and Design, Trials and Statistics (DTS) and are based across two sites in Sheffield City Centre.

#### Recruitment

A convenience sample of employees was recruited via an email inviting participation in the study. It was from this sample that volunteers were also obtained to participate in the intervention development.

#### Data collection

Data were collected between July and August 2014. Data collection took place at two intervals via email and online questionnaires. At baseline, participants completed a questionnaire of quantitative study measures and a pre-intervention seven-day prospective sitting log (sent and returned via email). The intervention began at week one (week beginning 7th July 2014) and ran over four weeks. At week five, a questionnaire of both quantitative and qualitative study measures and a post-intervention seven-day prospective sitting log were completed by participants.

### Measures

#### Quantitative data

All quantitative data were self-reported. Demographic information (age, gender, ethnicity, education), lifestyle factors (smoking, diet, alcohol consumption), and physical activity (using items derived from the International Physical Activity Questionnaire (IPAQ) [53] relating to time spent walking, or in moderate or vigorous activity respectively) were collected using a pre-intervention questionnaire only, consistent with previous studies looking at interventions to reduce sitting time [41, 54]. Workplace sitting time was assessed at baseline and the week immediately post-intervention (week five) using a seven-day prospective sitting log. A sitting log was used rather than an objective measure, as it provided a practical and less expensive alternative. There is a lack of validity and reliability data for assessing sitting time through the use of log-books, but to aid recall staff were asked to report the number of hours and minutes spent sitting at the end of each morning and afternoon work period.

Awareness of the various elements of the intervention was assessed at week five via the post-intervention questionnaire. Only participants who had not been involved in the intervention development were included in the subsequent analysis. Awareness was reported using the items: ‘were you aware that the intervention was running?’ (required a yes/no response); and ‘which elements of the intervention were you aware of?’ (required to select all of the following that applied: branding (logo), posters, emails, video links, links to reminder software, Twitter™ updates, information on the department homepage, workplace champions, management ‘leading by example’, I was not aware of the intervention, other).

#### Qualitative data

Qualitative data were obtained via open-ended questions as part of the post-intervention questionnaire in week five. The topics covered were: appropriateness of the intervention; effectiveness of the intervention; barriers to reducing workplace sitting not addressed by the intervention; other benefits of the intervention; and suggested improvements to the intervention.

### Analysis

#### Quantitative data

Statistical analyses were performed using Statistical Package for the Social Sciences (SPSS: An IBM Company, New York, USA) version 22. Demographic, lifestyle and physical activity data were presented as descriptive statistics (means ± standard deviations or rates/proportions multiplied by 100 to allow expression as percentages) for both the participants who completed all four elements of the data collection (the pre-intervention questionnaire and sitting log and post-intervention questionnaire and sitting log), i.e. “the completers”, and for those who only completed three or less elements of the data collection, i.e. “the non-completers”. Participation rates and rates of awareness of the intervention were also reported using descriptive statistics. Within group differences in daily workplace sitting time from baseline to post-intervention were presented using the mean ± standard deviation and confidence intervals.

#### Qualitative data

The qualitative data obtained from post-intervention questionnaires were thematically analysed. The themes were based on pre-determined categories: acceptability and feasibility of the intervention; impact; helpful elements; unhelpful elements; barriers that were not addressed by the intervention; other benefits that were realised; and suggested improvements to the intervention. The data were coded into raw data themes, which were allocated to the pre-determined categories for analysis.

## Results

### Intervention content

The intervention was drawn from the conceptualisation process and formative research and hence consisted of individual-, social-, organisational- and environmental-levels of influence. Table 1 highlights the suggestions obtained from the intervention development focus group and emails and Table 2 shows the final content of the intervention broken down by week and level of influence.

**Table 1:** Focus group and email suggestions for intervention content

• The use of prompts e.g. posters to encourage regular breaks placed by clocks in the office, posters to encourage drinking more water, and recommending the installation of free software onto computers to provide staff with an alternative way to remember to take breaks.	
• Carrying out walking and/or standing meetings.	
• Ensure management support in the form of encouraging emails and also “leading by example” e.g. by taking part in or initiating walking or standing meetings.	
• Provide an educational element to highlight the links between prolonged sitting and poor health – ideally with the use of You Tube™ videos.	
• Linking with a University of Sheffield wellness programme, which offers lunchtime exercise classes.	
• Developing a brand for the intervention to ensure that it is easily recognisable throughout ScHARR. The principal researcher determined the intervention brand, “Sit Less ScHARR!”, based on slogans from previous research e.g. "Stand-Up Victoria" [52]; "Stand-Up Australia" [28]; and "Stand-Up, Sit Less, Move More" [43].	
• The focus group participants volunteered to be “workplace champions” to encourage and support colleagues with the various elements of the intervention.	
• Utilising social media e.g. Twitter™ to promote the intervention.	
• Identifying low-cost environmental-level components was difficult. The only pragmatic suggestions were encouraging different uses of toilets/meeting rooms/printers /working spaces which were further away from their base.

**Table 2:** The final intervention

Level of Influence	Week 1	Week 2	Week 3	Week 4
Individual	• Weekly email (from management) containing: educational YouTube™ video and links to “reminder” software	• Weekly email (from management) with “Top Tips”	• Weekly email (from management) with “Top Tips” and link to the University’s wellness programme Health Checks	• Weekly email (from management) with “Top Tips”
Social	• Workplace champions promote stand-up/walking meetings/teaching sessions • “Incidental” walking - talking not emailing	• Lunchtime walks - supported by workplace champions	• Workplace champions promote standing/walking meetings	• Further lunchtime sessions (linked in with the University’s wellness programme) supported by workplace champions
Organisational	• Email sent from Dean to introduce the intervention, “Sit Less ScHARR!”	• Management support standing/walking meetings	• Management “lead by example” – take standing/walking meetings, regular breaks	• Final email from management to support improvements and advise they continue
Environmental	• Encourage using a different printer • Point of decision prompts: posters next to the lifts/office clocks	• Encourage working/having lunch in a different location • Point-of-decision prompts: posters encouraging standing/walking meetings	• Encourage meetings in a different location • Point-of-decision prompts: posters to drink more water	• Encourage using a different toilet

### Participation rates and sample description

**Fig. 1:**
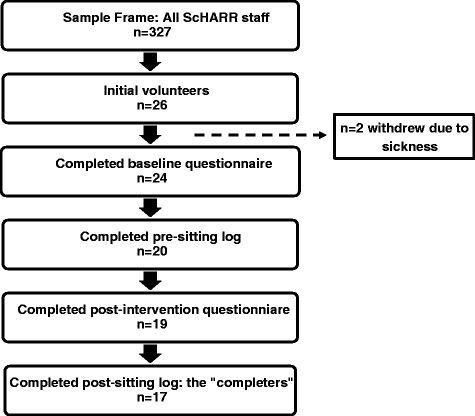
Flow chart showing participation and drop-outs at the various stages of the study *n* = 2 withdrew due to sickness

Of 317 staff, 26 (8.2 %) volunteered to participate in the evaluation of the intervention, two of whom withdrew due to sickness prior to data collection (see Fig. 1). Of the 24 participants, 17 (70.8 %) completed all data collection elements of the study. Only data obtained for these 17 "completers" were used for the subsequent statistical analyses i.e. missing data was not accounted for. The majority of "completers" were white (88.3 %), female (76.5 %) and all (100 %) had a postgraduate qualification (see Table 3). Furthermore, there was a wide variation in baseline physical activity participation, demonstrated by the large standard deviations around the number of days spent doing vigorous and moderate activity. More than a quarter (29.4 %) of "completers" participated in no vigorous or moderate physical activity. Participation in regular walking at baseline however was more consistent with all "completers" reporting walking during the previous seven days. Therefore, "completers" demonstrated a range of levels of physical activity. In addition, "completers" generally demonstrated positive health behaviours with 0 % being smokers, over 50 % eating five fruits or vegetables/day and almost 25 % not drinking alcohol.

**Table 3:** Baseline socio-demographic, workplace, sitting and activity characteristics "completers" and "non-completers"

Characteristic	Completers^a^	Non-completers^a^
Age:^b^		
25-34	47.1(8)	14.3(1)
35-44	41.2(7)	28.6(2)
45-54	5.9(1)	28.6(2)
55-64	5.9(1)	28.6(2)
Sex (M, F)	23.5(4), 76.5(13)	28.6(2), 71.4(5)
Ethnicity:^b, c^		
White English/Scottish/Welsh/Northern Irish/British	82.4(14)	100(2)^c^
White Irish	5.9(1)	0(0)
African	5.9(1)	0(0)
Chinese	5.9(1)	0(0)
Educational Attainment:^c^		
Higher education & professional/vocational equivalent	94.1(16)	100(2)^c^
Other (PhD)	5.9(1)	0(0)
ScHARR Department:^b^		
DTS	11.8(2)	0(0)
HEDS	41.2(7)	48.9(4)
HSR	11.8(2)	28.6(2)
PH	35.3(6)	28.6(2)
Base Building (Regent Court, BSI Building)	88.2(15), 11.8(2)	85.7(6), 14.3(2)
Contracted weekly working hours	35.6 ± 4.4	40.3 ± 6.9
Vigorous Physical Activity (VPA):		
Number of days spent doing VPA during the last 7 days	2.0 ± 1.7[29.4(5) did 0 VPA]	2.6 ± 2.3[28.6(2) did 0 VPA]
Length of each VPA session (minutes)	42 ± 36	51 ± 67
Moderate Physical Activity (MPA):		
Number of days spent doing MPA during the last 7 days	2.2 ± 2.3[29.4(5) did 0 MPA]	1.7 ± 2.6[57.1(4) did 0 MPA]
Length of each MPA session (minutes)	27 ± 22	17 ± 24
Walking:		
Number of days spent walking during the last 7 days	5.2 ± 2.1[100(17) did some walking]	5.3 ± 2.8[14.3(1) did 0 walking]
Length of each walking session (minutes)	61 ± 38	39 ± 31
Smoker (Y, N, Ex)	0.0(0), 88.2(15), 11.8(2)	14.3(1), 85.7(6), 0(0)
5-a-day (Y, N, Not sure)^b^	52.9(9), 29.4(5), 17.6(3)	71.4(5), 28.6(2), 0(0)
Number of units of alcohol consumed in 1 week:^b^		
0	23.5(4)	14.3(1)
1-2	0(0)	14.3(1)
3-4	17.6(3)	0(0)
5-6	29.4(5)	28.6(2)
7-8	17.6(3)	14.3(1)
9-10	5.9(1)	14.3(1)
>10	5.9(1)	14.3(1)

^a^Table presents %(n) or M ± SD

^b^Percentages may not add up to exactly 100 % due to rounding errors

^c^Ethnicity and educational attainment were part of the post-intervention questionnaire, which is why there were fewer participants who provided those data

### Comparisons of "completers" and “non-completers”

"Completers" and "non-completers" were of similar gender, ethnicity, educational attainment, base building, and smoking status (see Table 3). Age, ScHARR department and alcohol consumption were difficult to compare due to the small numbers and multiple categories. For baseline physical activity levels, "non-completers" participated in slightly more vigorous activity both in terms of number of days and duration of sessions, but slightly less moderate activity. "Completers" and "non-completers" reported walking for about the same number of days, but "non-completers" walked for a shorter duration. Due to the small number of "non-completers" (*n* = 7), statistical differences to "completers" were not assessed, but overall there appeared to be no substantial differences between the two groups.

### Behavioural response to the intervention

Time spent sitting in the morning both pre- and post-intervention was less than that in the afternoon by 12 and 5 min respectively (*p* = .411 and .782). Mean workplace sitting time was reported at 440 ± 79 min/day pre-intervention and 414 ± 80 min/day post-intervention (Table 4). Of the 17 "completers", 14 (82.4 %) reported a reduction in mean daily workplace sitting time post-intervention.

**Table 4:** Mean workplace daily sitting times pre- and post-intervention

	Mean am Pre-Intervention Sitting Time (mins)	Mean pm Pre-Intervention Sitting Time (mins)	Mean Daily Pre-Intervention Sitting Time (mins)	Mean am Post-Intervention Sitting Time (mins)	Mean pm Post-Intervention Sitting Time (mins)	Mean Daily Post-Intervention Sitting Time (mins)
Totals (M ± SD)	214 ± 42	226 ± 54	440 ± 79	205 ± 38	209 ± 55	414 ± 80

All time frames demonstrated a mean reduction in sitting time post-intervention (Table 5), with a mean difference in daily workplace sitting time from pre- to post-intervention of −26 ± 54 min/day (95 % CI = −2 to 53), which equated to a 6 % reduction.

**Table 5:** Difference in workplace sitting times pre- and post-intervention

	M ± SD	95 % CI
Difference in pre- and post-intervention am workplace sitting time (mins)	−9 ± 25	−4 to 22
Difference in pre- and post-intervention pm workplace sitting time (mins)	−17 ± 41	−4 to 38
Difference in pre- and post-intervention daily workplace sitting time (mins)	−26 ± 54	−2 to 53

### Awareness of the intervention

The post-intervention questionnaires provided data on the level of awareness of the intervention and its component elements. Participants who had been involved in the intervention development were excluded from the analysis. For the remaining participants, all (*n* = 13) were aware of the intervention as a whole and also the posters and emails, but there was a range of awareness for the other elements of the intervention from 69 % for the branding to 0 % for the workplace champions (Table 6).

**Table 6:** Awareness of the elements of the intervention amongst participants not involved in intervention development

Element of intervention	Aware %(*n*)
The intervention as a whole	100.0(13)
Posters	100.0(13)
Emails	100.0(13)
Branding (“Sit Less ScHARR!”)	69.2(9)
Reminder software	38.5(5)
Video links	30.8(4)
Management “leading by example”	15.4(2)
Twitter™ updates	15.4(2)
Information on ScHARR homepage	15.4(2)
Workplace champions	0.0(0)

### Participant views of the intervention

The qualitative data were taken from feedback provided in the post-intervention questionnaires (n = 19), which were analysed under the pre-determined categories. Selective and representative quotations to illustrate the categories are presented below.

#### Acceptability and feasibility

The responses to the intervention were generally positive and it was felt that the various elements of the intervention were practical. One participant stated that, “the suggestions made for [the intervention] fitted with what would be possible within a work environment like [this department]”.

An element where there was mixed feedback about the acceptability and feasibility was the standing/walking meeting. One participant felt that, “standing/walking meetings were less appropriate as most of the work I do involves leafing through results or looking at computer screens which works better with a desk and chair”. However, another participant stated that, “[this department] is quite a flexible work environment and so things like walking meetings would be acceptable”.

#### Impact

It was felt that the intervention made a positive impact on workplace sitting time due to:

- Awareness raising: “It made me more aware of the issue and made me think about how much time I spend sitting at work”.
- Reminders to sit less: “It kept the idea in my consciousness. It gave me ideas about things to try”.
- One regular staff meeting was converted to a standing meeting.

#### Most helpful elements of the intervention

There was a feeling that the intervention as a whole provided some generic positive changes within the department. One participant felt that the intervention resulted in “people talking about it - colleagues proposing walking meetings” and another felt that, “many people are receptive to the idea of moving more at the moment”.

Specific elements of the intervention, such as emails from management, were felt to provide a supportive culture to changing behaviour with one participant commenting that, “emails from the Dean showed support and reminded us that we should not feel guilty taking a break”.

Other helpful elements highlighted included:

- Standing/walking meetings: “Walking meetings both reduced sitting time and felt more efficient in terms of use of time”.
- Posters: “Posters created a feeling of community”.
- Reminder software/timers: “Using a timer to remind me to get up and move, walking a further distance to get a drink/photocopy etc.”.

One participant did not find any elements of the intervention helpful, stating, “I get uncomfortable sat down anyway, so I already do many of the suggestions”.

#### Least helpful elements of the intervention

Elements that were felt to be unhelpful for some participants included:

- Reminders: “I didn't want to put a reminder on my computer because I don't want to be distracted if I am in the middle of something that needs concentration”.
- Twitter™: “I don't use Twitter so this did not help for me”.
- Posters: “Posters - I was already up and about when I saw them!”.

#### Barriers to the intervention

Barriers that the intervention did not fully overcome included:

- The desk-based nature of work: “So much of my work relies on being sat at a computer which is unable to be addressed in a zero cost intervention”.
- Workload/time: “I was working to tight deadlines, so when engrossed with work, getting up and moving around weren't a priority”.
- Workplace environment: “Workspace not designed to promote reduced sitting time”.
- Views of peers: “Sometimes it looks like you're not working if you're not at your desk”.

#### Other benefits

Additional benefits of the intervention identified by participants included:

- Improved productivity/concentration: “Increased attention, if I got distracted I would move and then be able to concentrate fully again”.
- Reduced stress: “I found that leaving my desk for a short time reduced stress levels when working on difficult tasks”.
- Increased awareness of the associated health benefits: “Being more mindful and better informed of the health benefits of sitting less”.
- Improved workplace culture/changing social norms: “The intervention appears to have strengthened/increased the culture of working in more flexible and creative ways “.
- Improved physical health: “More energy from moving around more” and “less back ache”.

#### Improvements

The most commonly suggested improvement was to include ergonomic adaptations. Of particular note was the suggestion to utilise standing/treadmill desks which would allow a more substantial reduction in sitting time without disrupting workflow and productivity. One participant stated, “I guess for there to be a major improvement in sitting less there needs to be some workplace changes to really make sitting less possible”.

Other suggestions for improvement included:

- To integrate with other initiatives: “Perhaps it [the intervention] could have also been linked to #sitlessmovemore for more effective dissemination/reach and wider conversations”.
- More “leading by example”: “Senior staff/Meeting Chairs asked to introduce standing breaks in meetings”.
- Effective use of workplace champions: “Champions more visible. I didn't attend any meetings where people suggested standing, I think this should have been done more across the school”.
- Financial investment: “More money invested into practical solutions to encourage us to sit less yet maintaining productivity”.
- More scheduled activities: “If there was Tai Chi or yoga one-lunchtime a week in, say, [location close to their office], I'd definitely go”.
- Demonstrate elements are evidence-based: “Perhaps include sources on posters as someone mentioned on Twitter, to give them more credibility in an academic environment”.

## Discussion

This uncontrolled trial explored the acceptability and feasibility of a low-cost, co-produced, multi-modal intervention in reducing daily workplace sitting time amongst university desk-based workers. Three sources of information were used: pre-post behavioural responses; awareness of the various elements of the intervention; and views on the intervention.

Baseline sitting time of 440 min/day amongst study participants was higher than sitting time data for professional/managerial staff obtained in a previous study by Miller et al. [55] which looked at workplace sitting time by occupation. This could be explained by the differences in data collection: this present study used a sitting log, which was completed morning and afternoon by participants; whereas Miller et al. used the IPAQ. During this pilot, 82 % of “completers” reported a reduction in daily workplace sitting time, with a mean decrease of 26 min/workday (6 % reduction). Evaluating the effectiveness of this intervention was not an aim of this study, so it was not sufficiently powered to detect a statistically significant effect size (unless the effect size turned out to be very large); hence the quantitative findings need to be interpreted with caution. Nevertheless, the lack of statistical significance should not rule out potentially important benefits of the intervention. “Completers” were of comparable demographics to participants of similar studies [41–43, 56, 57] i.e. mainly White British females aged 35–44 years with a similar educational attainment and baseline level of physical activity.

On the whole, studies that evaluated multi-modal interventions that demonstrated statistically significant changes, reported a 10-25 % reduction in workplace sitting [41–43, 57]. The reasons for the reduction in sitting time in this study being lower than that of other multi-modal interventions may be explained by the lack of a true environmental element to this intervention such as sit-to-stand desks [41, 43, 57] or portable pedal machines [42]. Due to the need to develop low-cost interventions to support uptake amongst a variety of organisations, it was not considered appropriate to include such elements, which instead resulted in the development of a simple and pragmatic intervention.

The studies conducted by Neuhaus et al. [41] and Carr et al. [42] were based within a university setting similar to this pilot. A recent meta-analysis [58] reported 76 % of studies targeting improvements in health behaviours, such as physical activity, conducted in tertiary educational settings demonstrated significant health improvements and suggested that these settings should serve as a platform to research such intervention strategies. Nevertheless, findings from studies carried out in these settings may not be generalisable, as participants are not representative of the wider working population due to key demographic differences such as educational attainment and socio-economic status. Despite this, a study by Matei et al. [59], which looked at an intervention to reduce sitting time and increase physical activity amongst two different groups of older people, found different levels of uptake and impact amongst the different groups. Furthermore, a qualitative study by Bardus et al. [60], which looked at the reasons for participating and not participating in an e-health workplace physical activity intervention, demonstrated the importance of focusing on employees’ needs and motivators to behaviour change. These two studies highlight the importance of developing interventions that are tailored to the specific needs of that particular population and setting, rather than using a generic “one-size-fits-all” approach. Hence, an intervention developed for staff in a university setting for example may be different to an intervention developed for staff in a private sector small-to-medium sized enterprise.

Participants (not involved in the intervention development) were all aware of the intervention, but with some variation in the awareness of the different elements. There was a lack of awareness of some elements of the intervention such as the Twitter™ updates and the presence of workplace champions, which therefore rendered them unhelpful to those participants. The intervention as a whole was generally well-received by participants and was felt to be acceptable and feasible due to the ability of the different elements to be easily incorporated into the working day. It was felt that the intervention had a positive impact on workplace sitting as a result of awareness raising, reminders and support from management. Management support in particular was valued by participants in previous studies that evaluated similar interventions as a means of validating changes to health behaviour [41, 51]. There was a variety of feedback on the most helpful and least helpful elements, which seemed to be dependent on personal preference and awareness. Some participants felt that standing/walking meetings were helpful and easy to implement, whilst others felt that they were impractical and difficult to instigate. Barriers to reducing workplace sitting, which the intervention did not address, were highlighted and included: the nature of work; the workload/lack of time; attitudes of peers; and the workplace environment. The barriers highlighted by participants informed suggestions for improvements, which as far as possible need to be used to enhance further intervention development, highlighted by Neuhaus et al. [28] as good practice.

Aside from the reduction in workplace sitting, further benefits that were also described by participants, included: increased productivity, improved workplace culture, decreased stress, increased awareness of the health benefits of sitting less, and improved physical health. There is currently uncertainty in the literature regarding what the clinically important difference in sitting time needs to be in order to positively affect health. In a large population study [61], it was found that in the most sedentary, every hour/day increase in sitting time was associated with a 1.4 cm increase in waist circumference. Given that during this pilot daily, sitting time was reduced by an average of 26 min, it is possible that a change of this magnitude, if sustained, could result in positive health effects. However, determining health-related benefits was not an aim of this study and hence quantifiable data were not collected. Some studies have begun to evaluate whether multi-modal interventions to reduce workplace sitting correspond to improved health outcomes such as: anthropometric measures [43]; cardiometabolic risk factors [42, 43]; mood states [57]; and musculoskeletal symptoms [41, 57]. However, as a result of the short-term follow-up (4–12 weeks), mixed results have been reported. In addition, work-related outcomes, reported in this pilot as additional benefits, could be used to inform the argument for cost-effectiveness of such interventions. Nevertheless, determining work-related outcomes was not an aim of this study, so quantifiable data were not collected. Two studies evaluating multi-modal interventions [41, 43], which did include an assessment of work-related outcomes (self-rated work performance, absenteeism, presenteeism), did not observe statistically significant improvements. At present there is a dearth of evidence relating to the impact of such interventions on health- and work-related outcomes. Longer-term evaluations of similar interventions should include an assessment of these outcomes.

Since the completion of this pilot, a review by Gardner at el. [62] was published, which has highlighted the key elements of promising interventions to reduce workplace sitting. Firstly, the most promising interventions primarily aimed to change sedentary behaviour rather than physical activity. Secondly, this review found that interventions based on functions such as environmental restructuring and education, were most promising in reducing sedentary behaviour in the workplace. Finally, using behaviour change techniques such as self-monitoring of behaviour, adding objects to the environment, instruction on how to perform the behaviour, reviewing behavioural goals, providing information on health consequences, and behaviour substitution improved the promise of such interventions. Furthermore, the greater number of intervention functions and behaviour change techniques used, the more promising the workplace intervention. This pilot has many features of “promising interventions” as described by Gardner et al. including: the reduction in workplace sitting time as the primary aim; education being incorporated as a function of the intervention (e.g. awareness raising); utilising behaviour change techniques such as instruction on how to perform the behaviour (e.g. the use of prompts and reminder software to encourage regular breaks from sitting), providing information on health consequences (e.g. the educational You Tube™ video), and behaviour substitution (e.g. the use of standing/walking meetings). Despite this review by Gardner et al. being based on often low-quality evaluation methods, it has provided a good basis to improve future interventions aimed at reducing workplace sitting time. This pilot could usefully incorporate the findings of this review in any future work.

The main strength of this pilot was the adoption of a systematic and evidence-based intervention development process, incorporating conceptualisation and formative research. The fact that the intervention was grounded within a theoretical model (SEM) allowed the multiple levels of influence to be targeted, thereby ensuring that: individuals' autonomy and knowledge were increased; social networks were developed; and organisational support was obtained. The only element that this intervention unsatisfactorily addressed was the presence of true environmental changes. In addition, the use of a participatory approach has been demonstrated as an effective mechanism to reduce workplace sitting, which ensured a match between the needs of the staff and the suggested strategies [56]. The participatory approach to intervention development, ensured that the intervention was more likely to be acceptable to (and feasible for) staff. This approach allowed the development of a pragmatic intervention for use in a “real-world” setting. Finally, a further strength was the mixed methods approach, which ensured that the main aim of the study, assessing the feasibility and acceptability of a low-cost intervention, could be demonstrated, and has provided a basis for future research.

The major limitations of this pilot were the subjective nature of the data collection tool and the small number who provided individual data, relative to the number of staff invited to participate in data collection. The measurements of sitting time were based on self-report only, which may have introduced reporting and recall bias, although the prospective nature of the completion of the log may have minimised some of the bias.

Further limitations included:

1. The convenience sampling techniques used may have introduced selection bias. Participants may have been largely those already aware of the detrimental effects of prolonged workplace sitting or those who felt they might benefit the most from the intervention and hence were more committed to participating. Furthermore, the comparison between the “completers” and “non-completers” shows that, although there were no obvious differences between the two groups, it is unclear how representative the two groups were of the entire workforce. In addition, nothing was known about change in sitting time amongst the “non-completers”, so it is possible that “completers” were those who had a strong view about /were more aware of the intervention, which could have introduced further bias.
2. The small sample size and the nature of the intervention (such as posters and changes to meetings) made individual randomisation impossible and even cluster randomisation very difficult with likely contamination of any control group, meaning it was not possible to control for confounders. The lack of a control group, small sample size and probable selection bias may mean that the findings of this study are not widely representative of all ScHARR staff and hence are limited to the population studied and setting in which the study was conducted.
3. The lack of long-term monitoring of the effects of this pilot intervention along with the short intervention duration means that the potential for sustained reductions in workplace sitting have not been determined.

### Implications for policy and practice

This study has demonstrated the acceptability of low-cost interventions to reduce workplace sitting. The implications of such interventions on productivity at work requires further research, but the present evidence suggests that there is at least no decline in productivity or other work-related outcomes and it is possible that a reduction in sitting may improve productivity according to the findings from this study. Therefore, UK workplaces could consider pragmatic methods for reducing workplace sitting, which address multiple levels of influence and includes a participatory approach to ensure that the strategy is tailored to the needs of the workplace.

### Implications for future research

The findings of this pilot contribute to this emerging research paradigm and could be used to inform the development of a larger, cluster-randomised control trial. This more robust study design would allow for control of confounding and selection bias and has the ability to explore effect modification, whilst also producing a more precise estimate of effects. Furthermore, interventions tested over a greater duration including longer-term follow-up and objective monitoring techniques, to determine accurate and sustainable effects, are required. Any negative implications of such intervention and suggestions for intervention improvements should be incorporated into any further intervention development and evaluation ensuring the iterative nature of such a process. Including an environmental/ergonomic element within such multi-modal interventions is likely to yield more successful results, although will be more costly and hence may be a significant barrier to uptake amongst some employers.

Establishing whether there were differences between “completers”, “non-completers” and those who did not participate at all, may have provided further insight into the findings of this pilot and maximise the generalisability. Future research should ensure that everyone who is to be exposed to an intervention is recruited to participate in the study, not simply a self-selected sample as in this pilot. To support this, providing an incentive in return for participants’ time spent during the data collection process, in the form of either a financial incentive or simply feedback on how their sitting time compares with their peers, could be beneficial. In addition, conducting similar pilots in a variety of different sedentary workplaces (e.g. public sector, private sector, small-to-medium sized enterprises, and larger corporations) may provide a greater understanding of the steps that need to be taken to develop and evaluate successful interventions aiming to reduce workplace sitting, and hence also increase the generalisability of the findings.

This pilot has highlighted the importance of a systematic, evidence-based intervention development process, which should include a pilot study, so further research into interventions to reduce workplace sitting need to ensure a similar process is adopted. Furthermore, there is a requirement for future research to focus not only on whether an intervention successfully reduced workplace sitting, but on the impact such interventions could have on both health- and work-related outcomes. It is these findings which will allow a full assessment of the cost-effectiveness and hence sustainability of such interventions in UK workplaces.

## Conclusions

This study has reported findings relating to the acceptability and feasibility of an intervention to reduce daily workplace sitting of desk-based staff in an academic institution. It has provided support for using a participatory approach to inform intervention development, which is tailored to the individual workplace; the use of an evidence-based theoretical model such as SEM, which ensured multiple levels of influence were addressed; and the use of mixed methods to effectively evaluate such interventions. This pilot has provided a basis for future research and it is intended that this pilot be a pre-cursor to a larger controlled trial.

## Abbreviations

CI: Confidence interval
DTS: Design, trials and statistics section
HEDS: Health economics and decision science section
HSR: Health services research section
IPAQ: International physical activity questionnaire
M: Mean
MPA: Moderate physical activity
PH: Public health section
ScHARR: School of health and related research, University of Sheffield
SD: Standard deviation
SEM: Socio-ecological model
VPA: Vigorous physical activity
